# Dominant plant species shape soil bacterial community in semiarid sandy land of northern China

**DOI:** 10.1002/ece3.3746

**Published:** 2018-01-08

**Authors:** Shaokun Wang, Xiaoan Zuo, Xueyong Zhao, Tala Awada, Yongqing Luo, Yuqiang Li, Hao Qu

**Affiliations:** ^1^ Urat Desert‐grassland Research Station Northwest Institute of Eco‐Environment and Resources Chinese Academy of Sciences Lanzhou China; ^2^ Naiman Desertification Research Station Northwest Institute of Eco‐Environment and Resources Chinese Academy of Sciences Lanzhou China; ^3^ School of Natural Resources and Agricultural Research Division University of Nebraska‐Lincoln Lincoln NE USA

**Keywords:** 16S rRNA gene, dominant plant species, high‐throughput sequencing, Horqin Sandy Land, sand dunes

## Abstract

Plant species affect soil bacterial diversity and compositions. However, little is known about the role of dominant plant species in shaping the soil bacterial community during the restoration of sandy grasslands in Horqin Sandy Land, northern China. We established a mesocosm pots experiment to investigate short‐term responses of soil bacterial diversity and composition, and the related soil properties in degraded soils without vegetation (bare sand as the control, CK) to restoration with five plant species that dominate across restoration stages: *Agriophyllum squarrosum* (AS), *Artemisia halodendron* (AH), *Setaria viridis* (SV), *Chenopodium acuminatum* (CA), and *Corispermum macrocarpum* (CM). We used redundancy analysis (RDA) to analyze the association between soil bacterial composition and soil properties in different plant species. Our results indicated that soil bacterial diversity was significantly lower in vegetated soils independent of plant species than in the CK. Specifically, soil bacterial species richness and diversity were lower under the shrub AH and the herbaceous plants AS, SV, and CA, and soil bacterial abundance was lower under AH compared with the CK. A field investigation confirmed the same trends where soil bacteria diversity was lower under AS and AH than in bare sand. The high‐sequence annotation analysis showed that *Proteobacteria*,* Actinobacteria,* and *Bacteroidetes* were the most common phyla in sandy land irrespective of soil plant cover. The OTUs (operational taxonomic units) indicated that some bacterial species were specific to the host plants. Relative to bare sand (CK), soils with vegetative cover exhibited lower soil water content and temperature, and higher soil carbon and nitrogen contents. The RDA result indicated that, in addition to plant species, soil water and nitrogen contents were the most important factors shaping soil bacterial composition in semiarid sandy land. Our study from the pot and field investigations clearly demonstrated that planting dominant species in bare sand impacts bacterial diversity. In semiarid ecosystems, changes in the dominant plant species during vegetation restoration efforts can affect the soil bacterial diversity and composition through the direct effects of plants and the indirect effects of soil properties that are driven by plant species.

## 1 INTRODUCTION

Ecological restoration refers to the recovery process of degraded ecosystems to either the near pre‐ or preexiting state, in terms of ecosystem composition, structure, dynamics, and function (Aronson, Floret, Floc'H, Ovalle, & Pontanier, 1993; Davis & Slobodkin, 2004; Keenelyside, Dudley, Cairns, Hall, & Stolton, 2012). Restoration in protected areas focuses mainly on specific plant species composition and the reestablishment of local native vegetation (Keenelyside et al., 2012). Degraded sandy land restoration, through natural or human‐assisted efforts in northern China, emphasizes stabilizing shifting dunes with native vegetation (Zhao, Zhao, & Zhang, 2003), increasing plant species richness and diversity (Zuo et al., 2012), and improving soil nutrient content and organic matter (Li et al., 2008, 2015). Previous research on the restoration of degraded sandy land showed that both the dominant plant species and the vegetation composition shifted over time as expected (Zuo et al., 2012), and that soil fungal and macrofaunal diversity were driven by vegetation composition which increased across a successional restoration gradient from shifting dunes to semistabilized and stabilized dunes (Liu, Zhao, Zhao, Zuo, & Drake, 2009; Wang et al., 2016). In the case of microbial communities associated with vegetation, diverse plant communities have been shown to recruit microbial composition, either passively or actively (Mariadassou, Pichon, & Ebert, 2015), and plant diversity enhances the soil microbial diversity in the semiarid sandy land (Zuo et al., 2016).

The role of soil bacteria in terrestrial ecosystems is complex and critical to maintaining ecological function. Bacteria constitute over 90% of the soil microbial community, affecting species richness, and abundance (Rousk et al., 2010; Paul, 2014). They also play an active role in humus formation, litter decomposition, and material cycling, as well as soil structure development, plant nutrition, and performance (Van Der Heijden, Bardgett, & Van Straalen, 2008; Harris, 2009; Van Der Wal, Geydan, Kuyper, & de Boer, 2013; Cardinale, Grube, Erlacher, Quehenberger, & Berg, 2015; Jacoby, Peukert, Succurro, Koprivova, & Kopriva, 2017). Bacterial composition in soils can be associated with vegetation type and soil pH at both the local and continental scales (Kourtev, Ehrenfeld, & Häggblom, 2002; Fierer & Jackson, 2006; Lauber, Hamady, Knight, & Fierer, 2009; Zhalnina et al., 2015), and it is found to be closely impacted by environmental variability, such as soil nitrogen, carbon, moisture, texture, and structure, as well as human disturbance. (Vos, Wolf, Jennings, & Kowalchuk, 2013; Cederlund et al., 2014).

Although the role of microbes in soil processes is relatively well understood, the plant–microbe interactions and their associated role in enhancing plant performance have only recently started to be addressed (Jacoby et al., 2017). For example, it is unclear how the bacterial community responds to restoration efforts in semiarid grassland ecosystems and whether certain bacteria are only associated with specific host‐plant species. Such information is important to better understand the mechanistic processes associated with the plant–microbe interactions related to the establishment and performance of plants, which can then be used to improve and enhance the restoration efforts and ecosystem services of degraded lands.

Horqin Sandy Land is a typical semiarid area and is located in the southeastern part of Inner Mongolia, northern China. This area was once the most severely desertified region in northern China due to excessive reclamation and overgrazing (Wang, 2003; Zhao et al., 2015). Unsustainable land‐use practices in previous decades have shifted the ecosystem from its original savanna‐like landscape into degraded semiarid grasslands and bare shifting sand dunes (Zhao et al., 2003). The enforcement of the Fencing and Nongrazing Policy by the Chinese government accelerated the restoration of degraded sandy land since the 1970s. As a result, mobile dunes have gradually shifted into semistabilized and stabilized dunes and grasslands with different plant species began dominating across the different restoration stages of the sandy grasslands (Zuo et al., 2012; Zhao et al., 2015). In the past few years, an increasing number of studies on dune stabilization and vegetation restoration have been carried out in the Horqin Sandy Land (Zhao et al., 2014; Li et al., 2015; Zuo et al., 2015), including studies on fungal and macrofaunal community composition along the vegetation restoration gradients (Liu et al., 2009; Wang et al., 2016). However, the impacts of dominant plant species on the soil bacterial community remain unexplored in this region.

This study builds upon previously published studies (Wang et al., 2016; Zuo et al., 2016) and addresses two main hypotheses related to soil bacterial and plant interactions in restored grasslands: (i) Soil bacterial diversity and composition are impacted by dominant plant species; and (ii) soil factors are important in determining soil bacterial community structure in restored grasslands. To address these hypotheses, we investigated soil bacterial diversity and community structure, as well as soil properties, under different dominant plant species from different restoration stages of sandy grasslands. We used Illumina high‐throughput sequencing to assess soil bacterial community composition, determine whether specific bacterial taxa are associated with selected host plants in the short term, and identify soil factors that determine bacterial community.

## 2 MATERIALS AND METHODS

### 2.1 Study area

**Figure 1:**
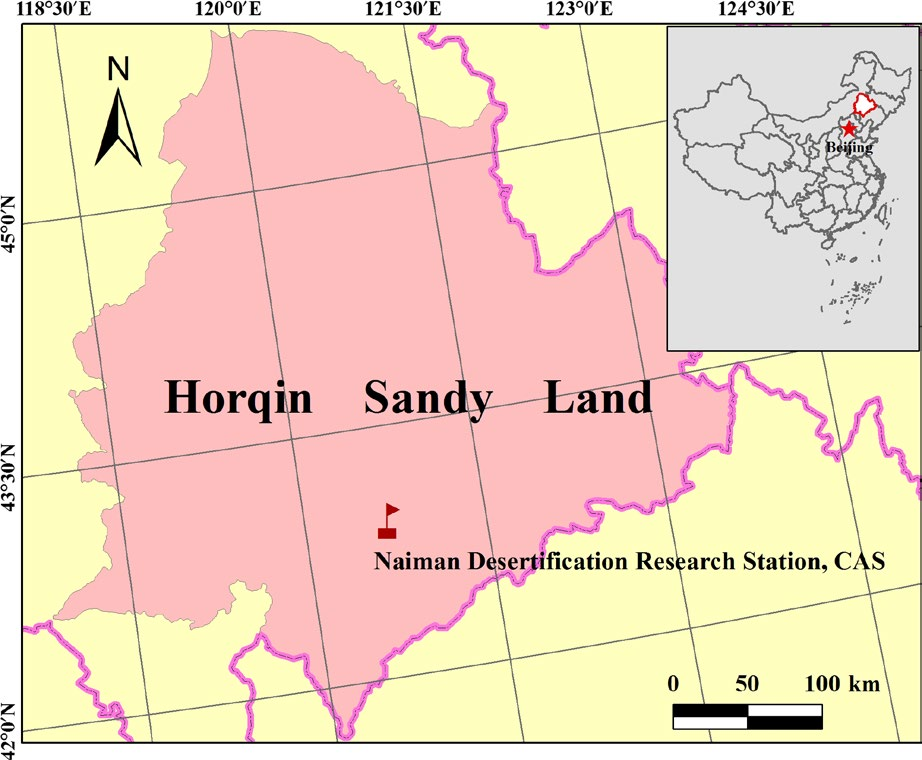
Location of the study site at Naiman Desertification Research Station, Chinese Academy of Science (CAS), in the Horqin Sandy Land of southeastern part of Inner Mongolia

The experiment was conducted at the Naiman Desertification Research Station (herein Naiman Station), Chinese Academy of Sciences (42°55′50″N, 120°41′51″E; al 360 m; Figure**** 1). The climate is characterized as temperate, semiarid continental monsoonal. The long‐term annual average precipitation is approximately 366 mm, of which 80% occurs during the growing season between June and September. The average annual potential evapotranspiration is 1935 mm. The average temperature is 6.4°C, ranging between a monthly maximum average of 24°C during the summer and minimum of −17°C in the winter. The soil is classified as sandy chestnut, which is light yellow in color and loose sand in texture, leading to its vulnerability to wind erosion (Li, Zhang, Zhang, & Shirato, 2003; Zhao et al., 2003; Wang et al., 2016). The native plant community dominating the area includes *Agriophyllum squarrosum* L., *Setaria viridis* L., *Artemisia halodendron* Turcz., *Caragana microphylla* L., *Melissitus ruthenicus* L., *Lespedeza davurica* L., *Cleistogenes squarrosa* L., *Corispermum macrocarpum*,* Chenopodium acuminatum,* and *Pennisetum centrasiaticum* Tzvel. (Zuo et al., 2012).

### 2.2 Experimental design

A flat sandy field was selected near Naiman Station, and 30 plastic pots (mesocosms, 60 cm height × 50 cm diameter) were buried in the field. Four layers of soils (0–10, 10–20, 20–40, and 40–60 cm) were separately taken from the mobile dunes and sieved at 2 mm to keep the soil homogenous across the layers. The pots were then filled with sieved soils in the sequence corresponding to each depth. The same amount of soil was added to each pot for each soil layer to minimize variability. We selected five native local plant species for this study: *A. squarrosum* is a pioneer annual herb that occupies mobile dunes; *A. halodendron* is a native shrub that dominates semistabilized dunes; *C. acuminatum* is one of the dominant herbaceous species in stabilized dunes; *C. macrocarpum* is one of the dominant herbaceous species in sandy grasslands; and *S. viridis* could be found in most habitats across the sandy land (Table S1). Species were planted randomly in the 30 pots, with five replicates for each plant species, leaving five pots without any plants as the controls (CK).

Seeds were collected from nearby natural vegetation at Naiman Station in August and September 2014. The seeds were soaked in sterile water for 30 min and sterilized under UV light for 20 min before sowing. Approximately 100–150 seeds were sown in each pot on 21 May 2015. Sufficient water (the same volume) was added twice to each pot to ensure the germination of the seeds immediately after sowing and at the end of May. Pots were weeded every 5 days to ensure that only the targeted plant species was growing in each pot. Seedlings were thinned out to 10–15 individuals in each pot to avoid competition. Soil samples were collected on 11 September 2015. A pooled sample was derived from five cores at a depth of 0–10 cm in each pot using the “S” collection pattern. The soil samples were sealed in sterile Ziploc bags and transported to the laboratory in an icebox for immediate analysis.

A complementary field study was established where rhizospheric soil samples were collected from a nearby site to compare to the mesocosm pot experiment. We selected five mobile dunes, in which both *A. squarrosum* (AS) and *A. halodendron* (AH) native plants were present. Rhizospheric soils from 10 to 15 plants per species were carefully collected within 0.5 mm of the plant roots for analysis. Bare soil samples were also taken at a depth of 0–10 cm in the mobile dunes as a control.

Soil parameters, including soil pH, electrical conductivity (EC), soil temperature (ST), soil water content (SWC), carbon (C), nitrogen (N), and particle size distribution were analyzed in each pot to assess their relationship with the soil bacterial community. Soil temperature (ST) and soil water content (SWC) were measured by a portable electrical thermometer and a soil moisture sensor, respectively. Soil particle size distribution was measured using the wet sieving method from the international and USDA classification systems. Soil pH and EC were determined in a 1:1 and 1:5 soil–water extract using a pH and EC probe, respectively. Soil total carbon (C) and nitrogen (N) were analyzed using an elemental analyzer (Elementar, Germany).

### 2.3 DNA extraction and sequencing

Total genome DNA was extracted from the soil samples upon their arrival to the laboratory, using the MoBio PowerSoil DNA Isolation Kit (MOBIO Laboratories, USA). The extracted genome DNA was stored at −80°C until sequencing. PCR amplification was conducted using the universal primer 515F‐806R, targeting 16S rRNA of V4 genes (Caporaso et al., 2011). PCR products were conducted using electrophoretic detection in 2% agarose gel. We mixed the qualified PCR products in equidensity according to their concentrations, and a Qiagen Gel Extraction Kit (Germany) was used for the purification of qualified PCR products. To build sequencing libraries, a TruSeq DNA PCR‐Free Sample Preparation Kit (Illumina, USA) was used according to manufacturer's instructions. The sequencing library was assessed by Qubit (Qubit 2.0 Fluorometer, Thermo Scientific) and Q‐PCR (Agilent Bioanalyzer 2100). Then, the sequencing was performed from the qualified library on the Illumina HiSeq2500 PE250 platform.

### 2.4 Data analysis

The primer and the barcode sequence were cut off from the 250 bp paired‐end reads generated from the Illumina platform. Raw tags were merged and assembled using FLASH (version 1.2.7; Magoc & Salzberg, 2011). In addition, high‐quality clean tags were obtained by filtering the raw tags using the quality controlled process of QIIME (version 1.7.0; Caporaso et al., 2010). To determine chimera sequences, the clean tags were aligned by the method that compares the UCHIME algorithm to the Gold Database (http://drive5.com/uchime/uchime_download.html), in order to find out chimera sequences. Finally, the chimera sequences were removed to develop the effective tags (Edgar, Haas, Clemente, Quince, & Knight, 2011).

Sequences from the effective tags were analyzed using UPARSE software (version 7.0.1001; Edgar, 2013). High similarity (≥97%) sequences were assigned to the same operational taxonomic unit (OTU). The representative sequence for each OTU was used for species annotation based on the Greengene Database (http://greengenes.lbl.gov/cgi-bin/nph-index.cgi) using the RDP (Ribosomal Database Project) (version 2.2) classifier algorithm (Wang, Garrity, Tiedje, & Cole, 2007). The phylogenetic relationships of the different bacterial species were aligned using the MUSCLE software (version 3.8.31).

Soil bacterial abundance (*A*) refers to the number of total effective tags in each sample. The observed species richness (*S*) is the number of OTUs. The ecological Shannon index (*H*) is used to determine the soil bacterial diversity.

$$H=\sum_{i=1}^{S}\frac{N_{i}}{A}\ln\frac{N_{i}}{A}$$

where *S* is the observed OTUs in each sample, *A* is the number of total effective tags in each sample, and *N*_*i*_ is the number of tags in the OTU_*i*_.

The bacterial diversity index is expressed as mean ± *SE* (*n *= 5). Significant differences among plant species and treatments were assessed by ANOVA and LSD tests at *p *< .05. Correlations between the bacterial community and soil factors were analyzed by Pearson two‐tailed tests. Origin (version 8.0) and PASW (version 18.0) were used to analyze the descriptive statistical data, significance tests, and correlations. The relative contribution of the soil factors to bacterial community composition was determined using ordination analysis. Detrended correspondence analysis (DCA) was first used to analyze the bacterial community, suggesting that redundancy analysis (RDA) was suitable for environment–community relationship analysis (length of gradient < 3; Liu, Zhao, Zhao, & Liu, 2015). The significance of the RDA correlations was determined by a Monte Carlo test. CANOCO (version 4.5) was used to conduct the DCA and RDA.

## 3 RESULTS

### 3.1 Effect of dominant plant species on soil bacterial abundance, richness, and diversity

**Figure 2:**
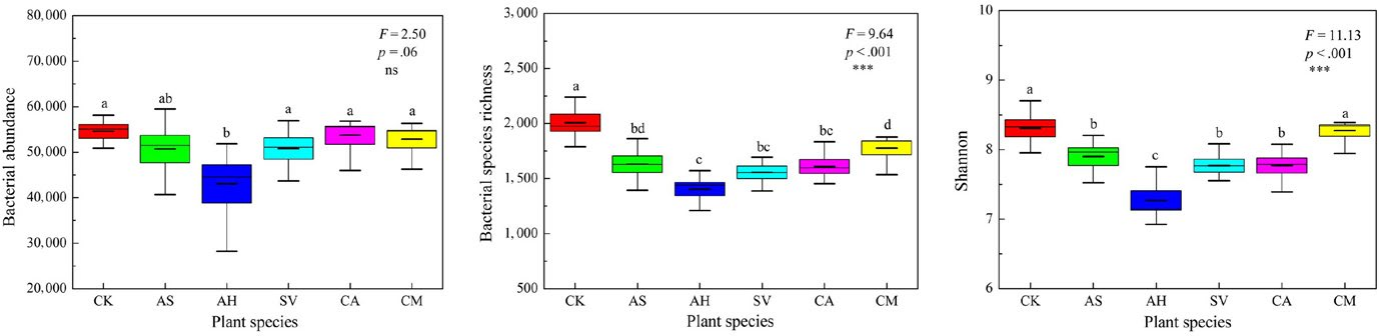
Soil bacterial abundance, richness, and Shannon diversity index as impacted by different plant species: *Agriophyllum squarrosum* (AS)*, Artemisia halodendron* (AH), *Chenopodium acuminatum* (CA), *Setaria viridis* (SV)*, and Corispermum macrocarpum* (CM), in Horqin Sandy Land, northern China

**Figure 3:**
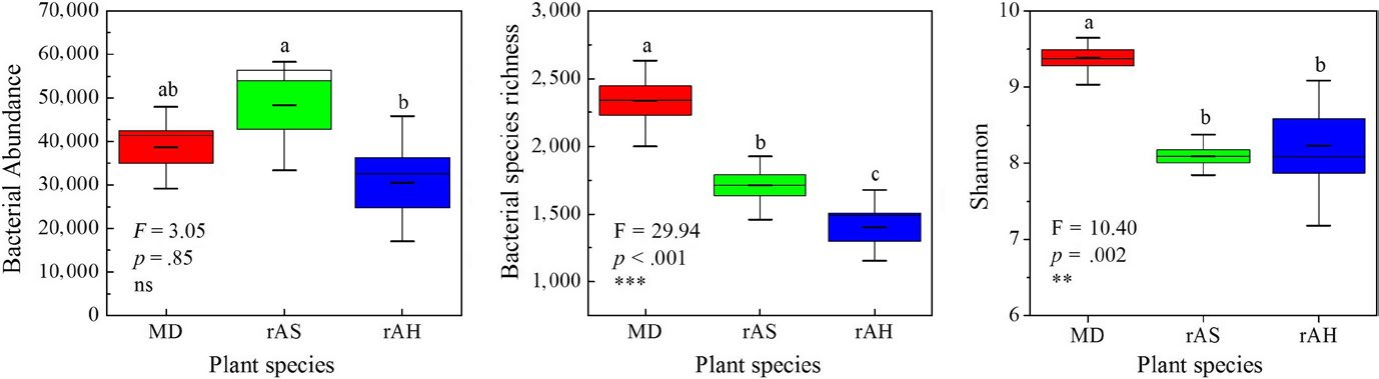
Field rhizospheric bacterial abundance, richness, and Shannon diversity index in mobile dunes. MD: mobile dunes, rAS: rhizospheric soil of *A. squarrosum*, rAH: rhizospheric soil of *A. halodendron*

The OTUs were obtained from the sequences with 97% similarity, and the statistical parameter of the OTUs was used to calculate soil bacterial abundance, richness, and diversity. Soil bacterial abundance, richness, and Shannon diversity were higher in bare sand (CK) than in planted pots (Figure**** 2). Bacterial abundance (*A*: the number of effective tags) was significantly lower in soils with the perennial shrub AH than in CK and in other plant species except for AS. Species richness (*S*) was significantly higher in CK soils than in those under the five dominant plant species, and it was the lowest under AH (*p *< .001). The highest Shannon diversity index (*H*) was recorded in CK soils (*H *= 8.31), and it was significantly higher than in planted soils, except for CM. The Shannon diversity index was the lowest under AH soils (*H *= 7.27). The results from the complementary field rhizospheric bacterial diversity study were consistent with those from the mesocosm pots experiment. Bacterial species richness and Shannon diversity index were significantly higher in bare sand of mobile dune (MD) than those in the rhizospheric soils of AS and AH (Figure 3).

### 3.2 Effect of dominant plant species on soil bacterial community structure

**Figure 4:**
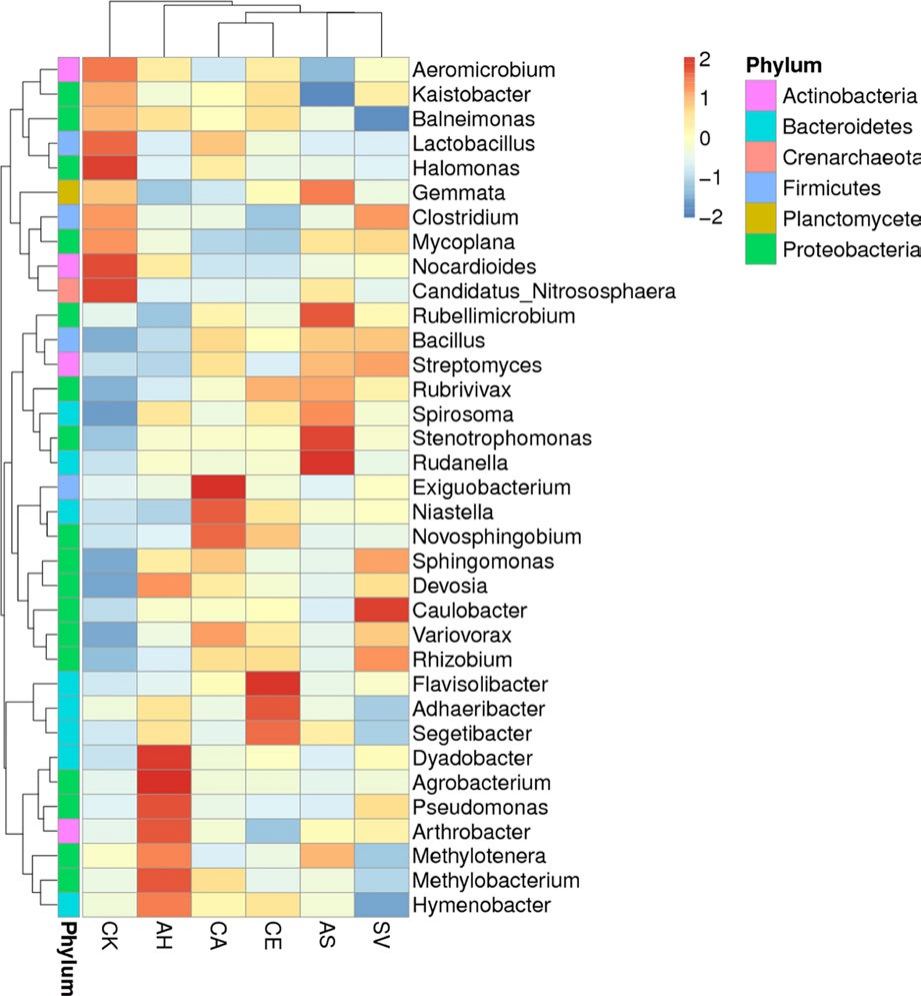
Cluster heatmap of bacterial community composition at the genus level

The bacterial species annotated by the OTUs were analyzed to determine the community structure. Table S2 summarizes the most abundant taxonomic groups in the soils of the five different plant species and CK at the phyla, class, and order levels. A cluster heatmap of the soil bacterial composition clearly showed the differences between the bacterial annotations at the genus level (Figure**** 4). According to the annotated OTUs, *Proteobacteria*,* Actinobacteria,* and *Bacteroidetes* represented the most abundant phyla in all samples, comprising 27.12–35.75%, 19.52–32.38%, and 20.75–34.01%, respectively, of the total OTUs in each treatment. These three phyla occupied more than 80% of each sample, and they did not show any significant difference among the plant species.

**Figure 5:**
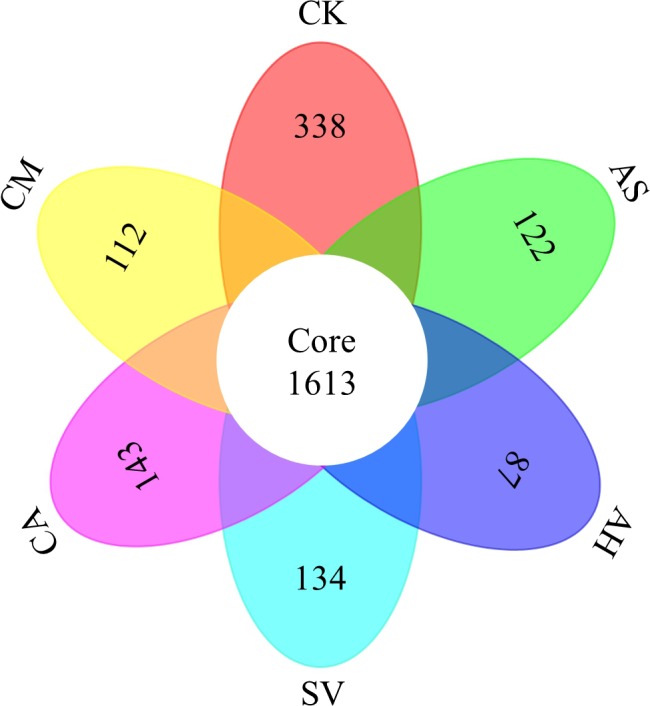
Venn diagram of the soil bacterial OTU associated with different plant species. The number of core represents the common OTU detected in all of the samples, and the numbers in different oval represent the specific OTU in different plant species

The Venn diagram represents the number of specific bacterial species (represented by OTU) associated with different plant species (Figure**** 5). The common bacterial species represented more than 90% of the total species recorded. However, a small percentage (5–10%) of specific bacterial species emerged in different host plants.

### 3.3 Effect of dominant plant species on soil properties

Plant species composition did not have a significant impact on soil pH, electrical conductivity (EC), or soil particle size distribution during the study. However, it significantly impacted the soil water content (SWC), temperature (ST), carbon (C), nitrogen (N), and C:N ratio (Table**** 1). SWC and ST were significantly higher in bare sandy soils (CK) than in the mesocosm pots with different plant species (*p *< .001). Soil total C and N increased significantly after planting (*p *< .05).

**Table 1:** Soil properties in bare sandy soil (control, CK), and under five different dominant plant species: *Agriophyllum squarrosum* (AS)*, Artemisia halodendron* (AH), *Chenopodium acuminatum* (CA), *Setaria viridis* (SV)*, Corispermum macrocarpum* (CM), in Horqin Sandy Land, China

Plant species	Particle size distribution			SWC (v%)	ST (°C)	pH	EC	C (%)	N (%)	C/N
	Coarse sand (0.1–2 mm)	Fine sand (0.05–0.1 mm)	Silt and clay (<0.05 mm)							
CK	95.86 ± 0.50 a	1.88 ± 0.21 a	2.26 ± 0.55 a	7.02 ± 0.27 a	26.13 ± 0.13 a,c	7.68 ± 0.10 a,b	8.93 ± 0.53 a,b	0.197 ± 0.017 a	0.054 ± 0.003 a	3.64 ± 0.19 a
AS	95.82 ± 0.61 a	2.02 ± 0.24 a	2.16 ± 0.52 a	5.93 ± 0.05 b,c	25.45 ± 0.15 b	7.75 ± 0.23 a	10.15 ± 0.72 a	0.255 ± 0.003 b	0.055 ± 0.002 a	4.63 ± 0.14 b
AH	95.66 ± 0.26 a	2.23 ± 0.10 a	2.02 ± 0.23 a	6.28 ± 0.04 b,d	25.69 ± 0.10 a,d	7.50 ± 0.03,b	9.58 ± 0.77 a,b	0.248 ± 0.009 b	0.069 ± 0.003 b	3.63 ± 0.29 a
SV	96.28 ± 0.28 a	1.82 ± 0.18 a	1.90 ± 0.16 a	5.92 ± 0.06 b,c	25.16 ± 0.04 c	7.61 ± 0.05 a,b	8.13 ± 0.40 b	0.254 ± 0.007 b	0.057 ± 0.002 a,b	4.44 ± 0.07 b,c
CA	96.50 ± 0.32 a	1.86 ± 0.16 a	1.64 ± 0.22 a	6.25 ± 0.07 b	25.26 ± 0.06 c	7.55 ± 0.03 b	8.65 ± 0.42 a,b	0.268 ± 0.005 b	0.056 ± 0.002 a	4.83 ± 0.21 b
CM	95.88 ± 0.43 a	2.08 ± 0.40 a	2.04 ± 0.22 a	5.64 ± 0.06 c	25.87 ± 0.07 b,d	7.67 ± 0.07 a,b	8.58 ± 0.16 a,b	0.253 ± 0.005 b	0.069 ± 0.009 b	3.85 ± 0.38 a,c
*F*	0.58	0.63	0.37	14.98	13.40	1.76	1.86	8.02	2.77	4.95
*p*	.72	.68	.86	<.001	<.001	.16	.14	<.001	.04	.003

### 3.4 Relationship between soil bacterial community and soil properties

**Figure 6:**
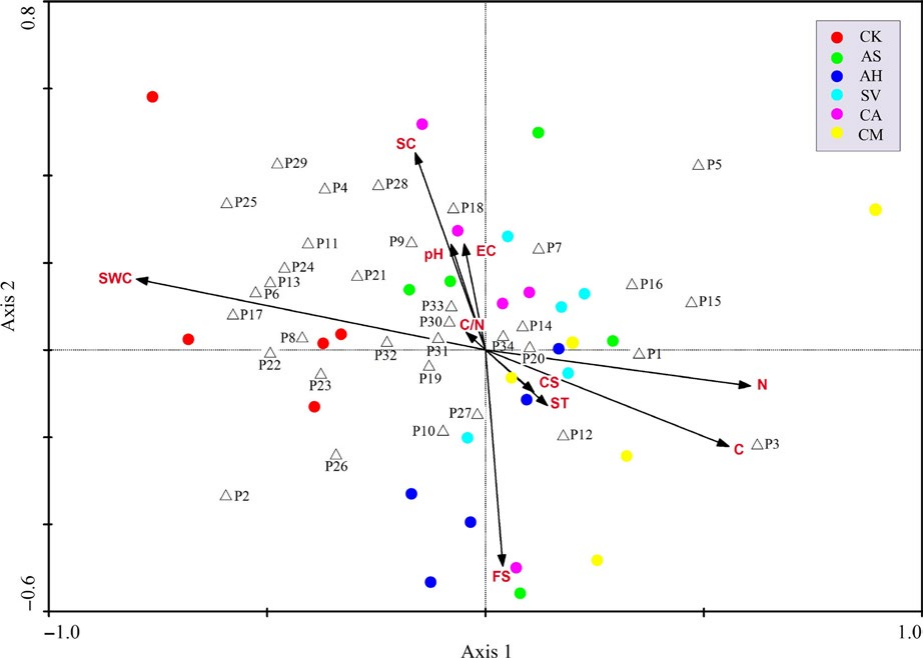
Two‐dimensional RDA ordination diagram for the first two axes, showing the distribution of the 30 samples (circles), 34 predominant bacterial taxa (triangles), and soil variables (vectors). Abbreviations of the bacterial taxa are the same as in Table4

RDA showed the contributions of the tested soil factors to the bacterial community composition as it was affected by different dominant plant species in sandy grasslands. The Monte Carlo test indicated that all of the canonical axes were significant (*p *= .026, Figure**** 6). Table**** 2 shows the correlations between the soil variables and the four axes of the RDA. The cumulative percentage variances of first and second axes were 63.1% and 19.3%, respectively, indicating the soil bacterial community was significantly affected by the measured variables. Specifically, the first axis was significantly and positively correlated with the soil carbon and nitrogen and negatively correlated with the soil water content (Table**** 2). The contribution of soil factors to soil bacterial community composition showed that only soil water content (*p *= .002) and soil nitrogen (*p *= .03) were significant under the Monte Carlo permutation test (Table**** 3), suggesting soil water content and nitrogen are the main soil factors that influence the bacterial composition associated with dominant plant species in degraded sandy grasslands.

**Table 2:** Intra‐set correlations of soil factors, eigenvalue, cumulative percent variance of bacterial species, and bacterial species‐environment correlation coefficients for the first four axes of RDA

Soil variables	Axis			
	SPX1	SPX2	SPX3	SPX4
C	0.414^a^	−0.150	−0.310	0.096
N	0.451^a^	−0.056	0.354	0.019
C:N	−0.033	0.027	−0.535^b^	0.096
CS	0.082	−0.065	−0.221	0.196
FS	0.029	−0.335	0.012	−0.041
SC	−0.119	0.306	0.258	−0.208
SWC	−0.594^b^	0.110	0.065	−0.012
ST	0.105	−0.086	0.240	0.102
pH	−0.059	0.164	0.022	−0.186
EC	−0.036	0.164	0.020	−0.188
Eigenvalue	0.286	0.087	0.056	0.013
Cumulative percentage variance %	63.1	82.3	94.7	97.6

C: soil carbon; N: soil nitrogen; CS: coarse sand (>0.25 mm); FS: fine sand (0.25–0.05 mm); SC: silt and clay (<0.05 mm); SWC: soil water content; ST: soil temperature; pH: soil pH value; EC: electrical conductivity. Abbreviations of the soil variables are the same blow.

a Correlation was significant at the 0.05 level (2‐tailed).

b Correlation was significant at the 0.01 level (2‐tailed).

**Table 3:** Redundancy analysis (RDA) of taxa data for quantifying the conditional effects of measured soil factors on bacterial taxa composition at the phylum level, using forward selection with Monte Carlo permutation test

Soil variables	Initial conditional effects	MCR (%)	*F*	*p*
SWC	0.186	18.6	6.41	.002**
N	0.127	7.9	2.89	.03*
C	0.110	4.7	1.4	.24
C/N	0.047	3.4	1.25	.29
SC	0.040	3.6	1.81	.13
ST	0.019	2.5	0.97	.41
pH	0.014	2.4	0.93	.42
EC	0.009	1.3	0.49	.67
CS	0.007	0.9	0.34	.84
FS	0.003	0	0.01	.98
Total		45.3		

Note: *the effect is significant at 0.05 level; **the effect is significant at 0.01 level.

The biplot demonstrated a clear separation (circles in different colors) between bare sand (CK) and planted pots. Total soil carbon and nitrogen increased along the first axis, and soil water content decreased. Correlations between soil bacterial taxa (triangles) and soil factors (vectors) were also shown in the biplot diagram (Figure**** 6). The results indicated that bacterial taxa responded differently to soil parameters (Table**** 4). Some taxa (P 2, 4, 6, 7, 10, 13, 15, 17, 21, 24, 25, 27, 29, 34) appeared to be positively correlated with soil water content, while others (P 4, 6, 10, 13, 17, 18, 21, 24, 26, 27, 28, 29, 33) were negatively correlated with soil nitrogen. Fewer taxa (P 2 and 5) were positively correlated with soil carbon, while some other taxa (P 2, 13, 27) were negatively correlated with soil carbon. P14 was positively correlated with soil temperature and pH, and P11 and P20 were only correlated with soil EC. Other taxa (P 1, 8, 9, 12, 16, 19, 22, 23, 30, 31) were weakly associated with the examined soil variables.

**Table 4:** Correlation coefficients between single bacterial taxa and soil variables

Bacterial taxa	Taxa number	C	N	CN	CS	FS	SC	SWC	ST	pH	EC
Proteobacteria	P1	−0.148	0.263	0.359	0.228	−0.013	−0.266	−0.208	−0.289	0.023	−0.086
Actinobacteria	P2	−0.365^a^	−0.304	0.036	0.098	0.067	−0.164	0.422^a^	−0.205	−0.046	−0.147
Bacteroidetes	P3	0.420^a^	0.221	−0.170	0.083	0.094	−0.164	−0.425^a^	0.045	−0.077	−0.116
Firmicutes	P4	−0.208	−0.443^a^	−0.138	−0.186	−0.206	0.364^a^	0.384^a^	−0.132	0.200	0.192
Cyanobacteria	P5	0.378^a^	0.203	−0.034	0.234	−0.316	−0.067	−0.284	−0.007	−0.233	−0.082
Acidobacteria	P6	−0.214	−0.379^a^	−0.117	−0.250	0.041	0.273	0.491^b^	0.048	0.234	−0.011
Crenarchaeota	P7	0.014	−0.185	−0.182	−0.340	0.170	0.294	0.462^a^	0.130	0.064	0.066
Verrucomicrobia	P8	−0.063	−0.210	−0.024	0.119	−0.158	−0.036	−0.152	0.059	0.114	−0.124
Planctomycetes	P9	−0.258	−0.154	0.170	0.073	−0.098	−0.021	0.153	0.228	0.233	0.060
Chloroflexi	P10	−0.189	−0.430^a^	−0.146	−0.221	−0.062	0.308	0.431^a^	0.286	0.430^a^	0.148
TM7	P11	0.217	0.156	−0.056	0.083	0.098	−0.166	−0.101	−0.022	−0.273	0.373^a^
Tenericutes	P12	0.027	0.118	0.036	0.045	0.029	−0.074	0.079	0.128	−0.147	−0.032
Gemmatimonadetes	P13	−0.402^a^	−0.380^a^	0.077	−0.018	−0.051	0.056	0.405^a^	0.096	0.327	−0.113
Euryarchaeota	P14	−0.028	−0.097	−0.041	−0.261	0.140	0.219	−0.030	0.471^b^	0.548^b^	0.250
FBP	P15	0.283	0.099	−0.115	−0.167	0.151	0.098	−0.398^a^	0.388^a^	0.368^a^	0.040
Fibrobacteres	P16	0.110	0.225	0.077	0.124	−0.178	−0.028	−0.226	−0.319	0.045	−0.281
Nitrospirae	P17	−0.194	−0.396^a^	−0.151	−0.232	0.027	0.261	0.517^b^	0.073	0.105	0.014
Armatimonadetes	P18	−0.215	−0.372^a^	−0.038	0.152	−0.270	0.001	0.217	−0.068	0.289	0.025
Thermi	P19	−0.156	−0.182	0.007	0.297	−0.058	−0.318	0.292	−0.337	0.056	−0.246
Fusobacteria	P20	0.336	−0.088	−0.339	−0.166	0.122	0.117	0.049	−0.085	−0.221	0.368^a^
Chlorobi	P21	−0.335	−0.565^b^	−0.174	−0.018	−0.222	0.173	0.522^a^	−0.089	0.234	−0.047
OP3	P22	−0.252	−0.352	−0.017	−0.014	−0.184	0.141	0.119	0.180	0.069	0.047
Chlamydiae	P23	−0.299	−0.190	0.127	−0.026	0.076	−0.020	0.261	−0.028	0.093	−0.097
WS3	P24	−0.319	−0.655^b^	−0.199	−0.102	−0.279	0.312	0.491^b^	0.092	0.301	0.051
Elusimicrobia	P25	−0.279	−0.325	−0.036	−0.113	−0.229	0.292	0.530^b^	−0.154	0.174	−0.019
GAL15	P26	−0.201	−0.388^a^	−0.108	0.128	−0.123	−0.071	0.122	−0.014	0.049	−0.105
Spirochaetes	P27	−0.414^a^	−0.436^a^	0.053	0.127	−0.511^b^	0.193	0.480^b^	−0.073	0.248	0.214
BHI80‐139	P28	−0.110	−0.469^b^	−0.270	−0.220	−0.036	0.289	0.357	0.181	0.200	−0.045
BRC1	P29	−0.309	−0.416^a^	−0.058	0.106	−0.126	−0.042	0.449^a^	−0.055	0.174	−0.242
TM6	P30	0.104	−0.134	−0.041	0.048	−0.150	0.043	−0.074	−0.135	0.024	−0.042
OD1	P31	−0.058	−0.346	−0.173	0.125	−0.173	−0.033	−0.089	0.175	0.009	−0.037
GN04	P32	−0.272	−0.058	0.213	0.339	0.039	−0.435^a^	0.150	−0.348	−0.057	−0.183
NC10	P33	−0.157	−0.387^a^	−0.073	0.062	−0.074	−0.024	0.196	0.032	0.145	0.050
AD3	P34	−0.335	−0.333	0.072	0.132	−0.154	−0.055	0.495^b^	−0.179	0.243	−0.067

a Correlation was significant at the 0.05 level (2‐tailed).

b Correlation was significant at the 0.01 level (2‐tailed).

**Figure 7:**
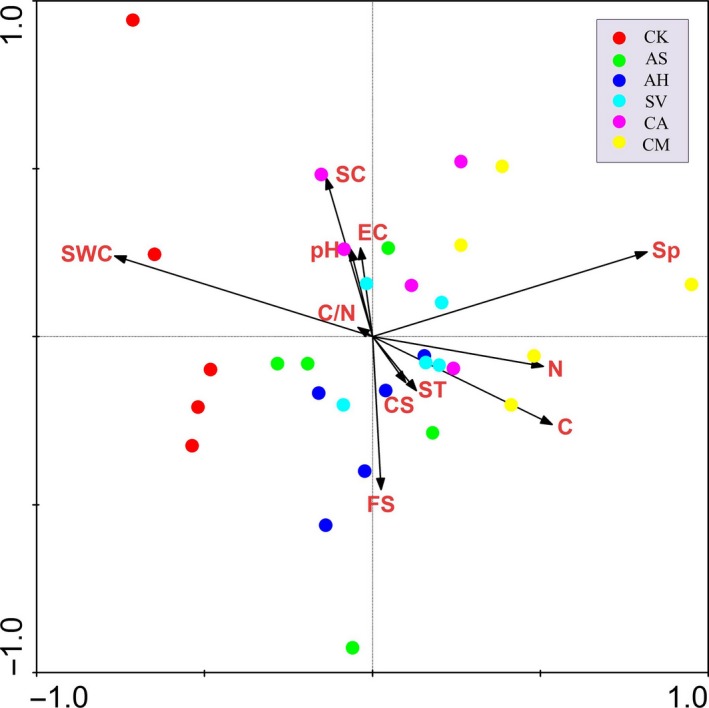
The two‐dimensional RDA ordination diagram for the first two axes, showing the distribution of the 30 samples (circles) and soil variables (vectors) with plant species (Sp) as an additional factor

When incorporating plant species as a parameter in the RDA, the results did not show a significant change in the distribution of the samples (circles in different colors) (Figure**** 7). The plant species (Sp, *p *= .002) became the most important factor that shaped the bacterial community structure, followed by soil total nitrogen (N, *p *= .04) and soil water content (SWC, *p *= .05).

## 4 DISCUSSION

During restoration efforts, plant species composition can shape the diversity and composition of soil microbial communities through root exudates in the rhizosphere and cause shifts in the soil environment, resulting in specific microbial populations colonizing certain host plants (Berg & Smalla, 2009; Prober et al., 2015). Results of this short‐term study indicated that, although the common bacterial species represented more than 90% of the total recorded species, a small portion (5–10%) of specific species emerged in different host plants. Bacterial species diversity and richness were higher in bare sandy soil (CK) than in planted soils; additionally, higher specific bacterial species were detected in CK compared with the vegetated soils. This could be due to shifts in the soil environment, especially in nutrient sources for bacteria, such as nitrogen and water, which are mostly driven by plant root exudates and physiological characteristics. These shifts favor certain bacterial species over others, in our case leading to the emergence of less diverse and more host‐specific communities associated with the plant rhizosphere. In our study, the soil and environmental conditions were similar before planting and the presence of vegetation‐modified soil factors over one growing season, shaping the bacterial community. Soil microorganisms are highly responsive to the quality of soil labile organic carbon substrate. Labile substrates are influenced by the natural input into the soils, and they are the main driving force for bacterial mineralization (Li et al., 2015). The five plant species were functionally different—with different growth habits and resource use strategies—leading to differences in the soil environment and bacterial community. For example, soil bacterial diversity was significantly lower under AH, a perennial shrub, relative to the bare sandy soils in both pot (mesocosm) and field rhizospheric experiment. This is probably due to the root morphology (greater root mass) and quality characteristics (higher lignin to cellulose ratio) in this perennial species relative to its annual counterparts or bare sandy soils (Luo, Zhao, Ding, & Wang, 2016). These results demonstrated that species selection must be taken into consideration during restoration efforts, and changes of vegetation cover that are caused by climate variability and extremes, as well as anthropogenic management can have significant impacts on soil microbial communities and soil health (Berg & Smalla, 2009).

Microbial diversity could be used to describe the biological organization and complexity at different scales, from genetic variability within and between taxa to functional groups in ecosystems (Torsvik & Øvreås, 2002). Bacterial functions can be assessed through their ecological activities; however, with the development of high‐throughput sequencing, it is now possible to identify gene characteristics in microbes and to better understand gene functions and metabolic pathways (Vanwonterghem, Jensen, Ho, Batstone, & Tyson, 2014; Cong et al., 2015). Some of the genes are associated with litter decomposition (Wang et al., 2015; Wymore et al., 2016), and others are associated with nutrient cycling (Paul, 2014), such as carbon (Nielsen, Ayres, Wall, & Bardgett, 2011; Schimel & Schaeffer, 2015) and nitrogen (Bru et al., 2011; He, Shen, Zhang, & Di, 2012; Tu et al., 2017). In this study, we detected some taxa that are closely related to various ecological functions. For example, Cyanobacteria (P5, occupied approximately 2% of total OTU) have been recognized for their oxygenic photosynthesis properties (Soo et al., 2014); Crenarchaeota (P7, 0.1–1%), associated with ammonia oxidation, have been identified as ammonia‐oxidizing archaea (AOA) (Leininger et al., 2006; Francis, Beman, & Kuypers, 2007; Smith, Casciotti, Chavez, & Francis, 2014); Nitrospirae (P17, 0.03–0.6%) is ubiquitous in terrestrial environments and plays a vital role in the nitrification of the nitrogen cycle (Martínez‐Alonso, Escolano Sánchez, Montesinos Seguí, & Gaju, 2010); Chloroflexi (P10, 1%) is a wide spread phylum, which is associated with nitrite‐oxidizing bacteria (NOB) (Sorokin et al., 2012) and granulation and preservation of the granule structure (Yamada et al., 2005); Chlorobi (P21, 0.05%), the members of which are physiologically similar to “green sulfur bacteria” (GSB) can produce chlorosomes as their light‐harvesting complexes (Liu et al., 2012). Although we detected a small fraction (<5%) of those specific functional bacteria, their ecological functions should not be underestimated. They are closely related to nutrient cycling, organic matter decomposition, and help regulate the development of soil structure and plant productivity in terrestrial ecosystems, especially in the N‐limited semiarid sandy grasslands.

Soil bacterial communities are sensitive to environmental changes. Many studies have shown that environmental factors, mainly pH and nitrogen, are the main predictors of soil bacterial diversity (Lindström & Langenheder, 2012; Zhalnina et al., 2015). Soil pH has been found to be significantly associated with soil bacterial community structure at the continental scale (Lauber et al., 2009) and in long‐term experiments (Zhalnina et al., 2015). Nitrogen is thought to be the key driver for changes in bacterial structure in agricultural soils (Cederlund et al., 2014). Soils are considered of the most heterogeneous in terrestrial ecosystem, due to variable labile organic matter input by plants within and across ecosystems (Kuzyakov & Blagodatskaya, 2015). Our results showed that soil properties shifted in responses to the plant species, and soil water content and nitrogen were the main factors that were significantly correlated with the soil bacterial community structure. On the other hand, we did not record any significant correlation between soil bacterial community and pH, since the later did not change significantly in response to vegetation presence during the one‐year experiment. Although soil water content does not play a significant role in the bacterial structure at the continental scale (Lauber et al., 2009), it may have a stronger effect in a semiarid areas when examined at the local scale.

## 5 CONCLUSION

Our findings clearly illustrated the associations among soil bacterial diversity and composition with plant species changes and plant‐induced soil properties in sandy grassland ecosystem. The bacterial species richness and diversity declined in vegetated plots compared with bare sandy soils. The dominant bacterial taxa were universal in soils with different plant species, and distinctive bacterial taxa associated with specific host plants were also detected. Soil properties could explain a significant proportion of variation in the bacterial composition. In addition to plant species, soil water and nitrogen contents were key factors in determining soil bacterial community structure. Changes in the dominant plant species in the restoration of sandy grassland not only directly determine soil bacterial diversity and composition but also alter the key soil properties to affect the soil bacterial community. These results address the role of changes in dominant plant species interacted with plant‐induced soil properties in shaping the soil bacterial community, thus providing the valuable support for the future studies of the plant–microbe–soil interactions in vegetation restoration of semiarid sandy grasslands.

## CONFLICT OF INTEREST

None declared.

## AUTHOR CONTRIBUTION

Shaokun Wang and Xiaoan Zuo conceived and planned the experiment. Xueyong Zhao developed the experimental design. Yongqing Luo, Yuqing Li, and Hao Qu participated in the field and laboratory work. Shaokun Wang performed the experiment and wrote the original manuscript. Tala Awada contributed the data analysis and manuscript editing. All authors contributed to the final manuscript.
