# Supplementary material for: Dominant plant species shape soil bacterial community in semiarid sandy land of northern China
Source: Ecol Evol. 2018 Jan 8;8(3):1693–704. doi: 10.1002/ece3.3746 (PMC5792618; doi:10.1002/ece3.3746)
Supplement: Supplementary file 1 [file ECE3-8-1693-s001.doc]

**Table S1.** Characteristics of the 5 dominant plant species used in the pot experiment in Horqin Sandy Land, northern China

| Plant species | Habitat | Photosynthetic pathway | Life form |
| --- | --- | --- | --- |
| *Agriophyllum squarrosum* (AS) | Shifting dunes | C4 | Annual herb |
| *Artemisia halodendro* (AH) | Semi-stabilized dunes | C3 | Shrub |
| *Setaria viridis* (SV) | All habitats | C4 | Annual herb |
| *Chenopodium acuminatum* (CA) | Stabilized dunes | C4 | Annual herb |
| *Corispermum macrocarpum* (CM) | Sandy grassland | C3 | Annual herb |

**Table S2.** Relative abundance of the predominant bacterial groups based on the 16S rRNA sequences analysis (%). Values less than 0.5% are not listed.

| Phylum | Class | Order | CK | AS | AH | SV | CA | CM |
| --- | --- | --- | --- | --- | --- | --- | --- | --- |
| Actinobacteria | Actinobacteria | Actinomycetales | 25.8 | 23.5 | 29.0 | 25.8 | 21.1 | 16.0 |
|  | Acidimicrobiia | Acidimicrobiales | 2.2 | 1.0 | 0.6 | 0.7 | 1.0 | 1.1 |
|  | Thermoleophilia | Solirubrobacterales | 1.9 | 0.7 | 0.6 | 0.8 | 0.9 | 1.2 |
|  |  | Gaiellales | 0.7 | 0.3 | 0.3 | 0.3 | 0.3 | 0.4 |
| Proteobacteria | α-proteobacteria | Caulobacterales | 0.6 | 0.5 | 0.5 | 1.1 | 0.4 | 0.4 |
|  |  | Rhizobiales | 3.7 | 4.3 | 4.9 | 5.8 | 5.0 | 4.9 |
|  |  | Rhodobacterales | 2.2 | 3.6 | 1.8 | 2.6 | 2.7 | 2.4 |
|  |  | Rhodospirillales | 0.7 | 0.8 | 0.9 | 0.7 | 0.7 | 0.8 |
|  |  | Sphingomonadales | 1.7 | 1.8 | 2.0 | 2.3 | 3.7 | 3.4 |
|  |  | Rickettsiales | 0.1 | 0.6 | 0.1 | 0.5 | 0.7 | 0.6 |
|  | β-proteobacteria | Burkholderiales | 10.4 | 12.9 | 15.0 | 14.8 | 16.0 | 12.2 |
|  |  | Methylophilales | 0.8 | 1.1 | 1.3 | 0.4 | 0.6 | 0.6 |
|  | δ-proteobacteria | Myxococcales | 1.3 | 1.3 | 0.8 | 1.1 | 1.1 | 1.3 |
|  |  | Bdellovibrionales | 0.4 | 0.4 | 0.5 | 0.5 | 0.4 | 0.5 |
|  | γ-proteobacteria | Pseudomonadales | 0.5 | 1.3 | 2.1 | 1.3 | 0.9 | 0.8 |
|  |  | Xanthomonadales | 0.6 | 1.4 | 0.6 | 0.9 | 0.8 | 1.1 |
| Bacteroidetes | Saprospirae | Saprospirales | 14.2 | 16.1 | 15.1 | 17.2 | 17.3 | 23.0 |
|  | Cytophagia | Cytophagales | 5.8 | 9.8 | 9.9 | 7.6 | 7.3 | 9.8 |
|  | Sphingobacteriia | Sphingobacteriales | 0.5 | 0.5 | 0.8 | 0.7 | 0.9 | 0.7 |
| Firmicutes | Bacilli | Bacillales | 1.0 | 0.9 | 0.7 | 1.5 | 1.6 | 1.1 |
|  |  | Lactobacillales | 4.2 | 0.1 | 0.1 | 0.1 | 2.8 | 0.8 |
|  | Clostridia | Clostridiales | 0.2 | 0.2 | 2.0 | 0.1 | 0.1 | 0.1 |
| Verrucomicrobia | Pedosphaerae | Pedosphaerales | 1.1 | 1.3 | 0.5 | 0.9 | 0.7 | 1.0 |
|  | Spartobacteria | Chthoniobacterales | 1.2 | 0.7 | 0.5 | 0.9 | 0.4 | 1.0 |
|  | Verrucomicrobiae | Verrucomicrobiales | 0.2 | 0.3 | 0.2 | 0.5 | 0.2 | 0.3 |
| Acidobacteria | Acidobacteria | iii1-15 | 0.7 | 0.3 | 0.2 | 0.2 | 0.3 | 0.4 |
|  | Chloracidobacteria | RB41 | 1.3 | 0.8 | 0.4 | 0.6 | 0.5 | 0.7 |
| Cyanobacteria | Chloroplast | Streptophyta | 0.1 | 1.0 | 0.2 | 1.3 | 2.7 | 2.6 |
| Planctomycetes | Planctomycetia | Pirellulales | 0.9 | 1.2 | 0.3 | 0.5 | 0.5 | 0.6 |
|  |  | Gemmatales | 0.5 | 0.6 | 0.1 | 0.2 | 0.2 | 0.3 |
| Crenarchaeota | Thaumarchaeota | Nitrososphaerales | 1.4 | 0.7 | 0.1 | 0.2 | 0.2 | 0.2 |
